# A New Natural Lactone from *Dimocarpuslongan* Lour. Seeds

**DOI:** 10.3390/molecules17089421

**Published:** 2012-08-06

**Authors:** Gongming Zheng, Xiaoyi Wei, Liangxiong Xu, Zhongjun Li, Gangyong Liu, Xiance Zhang

**Affiliations:** 1 Guangdong Food and Drug Vocational College, Guangzhou, Guangdong 510520, China; Email: lizj@gdyzy.edu.cn (Z.L.); liugy@gdyzy.edu.cn (G.L.); zhangxc@gdyzy.edu.cn (X.Z.); 2 South China Botanical Garden, the Chinese Academy of Sciences, Guangzhou, Guangdong 510650, China; Email: weixiaoy@scbg.ac.cn (X.W.); xlx048@scbg.ac.cn (L.X.)

**Keywords:** *Dimocarpuslongan* Lour., longan seed, constituents, lactone

## Abstract

A new natural product named longanlactone was isolated from *Dimocarpuslongan* Lour. seeds. Its structure was determined as 3-(2-acetyl-1*H*-pyrrol-1-yl)-5-(prop-2-yn-1-yl)dihydrofuran-2(3H)-one by spectroscopic methods and HRESIMS.

## 1. Introduction

Longan [*Dimocarpus* *longan* Lour. (syn. *Euphoria* *longana* Lam.)] is an evergreen tree of the Sapindaceae family, which is widely cultivated in Southern China, India, and Southeast Asia [1]. Longan fruit is one of the most favoured tropical fruits in China [2]. Longan seeds have long been used as a folk medicine in China for treatment of acariasis, hernia, wound hemorrhages, eczema and scrofula [3], which have recently also been proven to possess free radical-scavenging activity [4], antifatigue properties [5], growth inhibition of colorectal carcinoma cells [6], and hypoglycemic effects [7]. Longan seeds have been found to be a rich source of antioxidant phenolic compounds which are promising as functional food ingredients or natural preservatives. Soong and Barlow reported that longan seeds contained thirteen polyphenols, such as gallic acid, corilagin and ellagic acid [8]; recently Sudjaroen *etal.*, identified eleven polyphenolic compounds from longan seed, including chebulagic acid, ellagic acid 4-*O*-α-L-arabinofuranoside, isomallotinic acid and geraniin, *etc.* [9]. We have investigated the chemical constituents of longan seeds and succeeded in the isolation of eight polyphenols and twelve other compounds [10,11]. This paper deals with the isolation and structure elucidation of a new natural lactone product named longanlactone from longan seeds.

## 2. Results and Discussion

**Figure 1:**
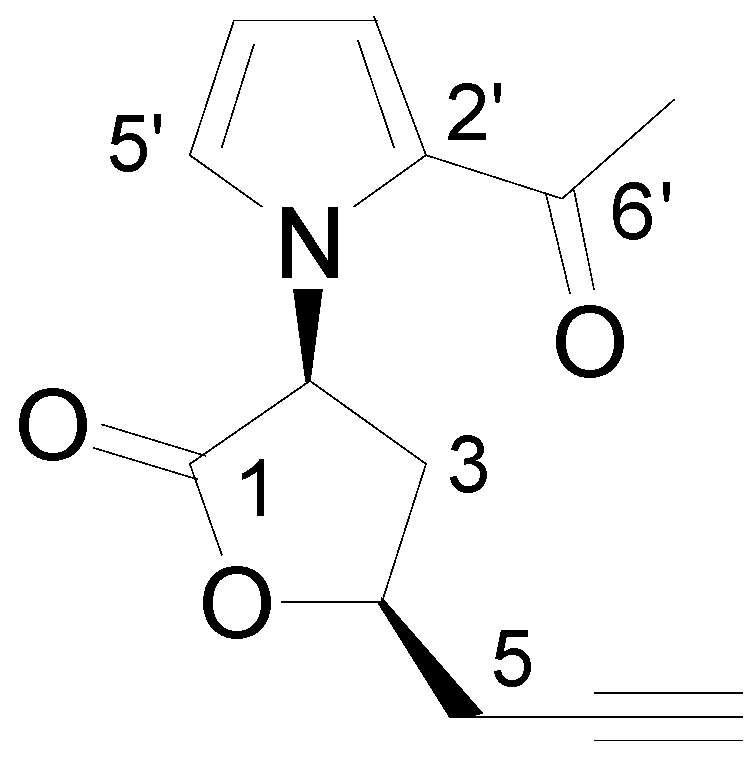
Structure of longanlactone.

The new compound (Figure 1) was obtained from the chloroform extracts of longan seeds as white acicular crystals with the melting point of 198–200 °C. The HR-ESI-MS spectrum revealed a quasi-molecular ion peak at 232.0971 [M+H]^+^ (calculated 232.0973) corresponding to the molecular formula of C_13_H_14_NO_3_.

Its UV spectrum exhibited peaks at 253 and 286 nm, which are characteristic bands of acetylpyrroles. The IR spectrum demonstrated the existence of alkynyl (3,306 and 2,111 cm^−1^) and carbonyl groups (1,770 and 1,640 cm^−1^). The ^1^H-NMR spectral data (Table 1) exhibited signals for 13 nonexchangeable protons, including three double doublets at δ 7.07 (1H, *J* = 4.0, 1.6 Hz), 6.26 (1H, *J* = 4.0, 2.6 Hz), 6.97 (1H, *J* = 2.6, 1.6 Hz) for a 2-pyrryl group, a singlet at δ 2.46 (3H, s) for a tertiary methyl group, a triplet at δ 2.07 (1H, *J* = 2.6 Hz) for a methine, a multiplet at δ 4.69 (1H) for an oxygenated methine, a broad singlet at δ 5.88 (1H) for a nitrified methine, four double double doublets at δ 2.97 (1H, *J* = 12.4, 9.2, 6.1 Hz), 2.37 (1H, *J* = 22.2, 12.4, 2.0 Hz), 2.89 (1H, *J* = 16.8, 5.3, 2.8Hz), 2.82 (1H, *J* = 16.8, 7.1, 2.8 Hz) for two methylenes. The ^13^C-NMR spectrum (Table 1) showed four aromatic carbons (δ 130.0, 121.4, 109.7, 129.7), of which one was a quaternary carbon (δ 130.0) for a 2-pyrryl group, two carbonyl carbons (δ 171.8, 188.7), four oxygenated and nitrified carbon or alkynyl carbon (δ 71.6, 77.2, 74.3, 58.2), of which one was a quaternary carbon (δ 77.2), two methylene carbons (δ 24.8, 35.5) and one methyl carbon (δ 27.0).

**Table 1:** ^1^H-NMR and ^13^C-NMR data of longanlactone in CDCl_3_ (*J* in Hz).

Position	^1^H	^13^C	HMBC	NOESY
1		171.8 s		
2	5.88 (br s)	58.2 d		3a, 3b, 4, 5'
3a	2.97 (ddd, 12.4, 9.2, 6.1)	35.5 t	1, 2	2, 3b, 4
3b	2.37 (ddd, 22.2, 12.4, 2.0)		2, 4, 5	3a, 4, 5b, 5'
4	4.69(m)	74.3 d	6	2, 3a, 3b, 5a, 5b
5a	2.89 (ddd, 16.8, 5.3, 2.8)	24.8 t	3, 4, 6, 7	4
5b	2.82 (ddd, 16.8, 7.1, 2.8)		3, 4, 6, 7	3b, 4
6		77.2 s		
7	2.07 (t, 2.6)	71.6 d	4, 5	
2'		130.0 s		
3'	7.07 (dd, 4.0, 1.6)	121.4 d	4', 5'	4', 7'
4'	6.26 (dd, 4.0, 2.6)	109.7 d	2', 3'	3', 5'
5'	6.97 (dd, 2.6, 1.6)	129.7 d	2', 3', 4'	2, 3b, 4'
6'		188.7 s		
7'	2.46 (s)	27.0 q	2', 3', 6'	3'

**Figure 2:**
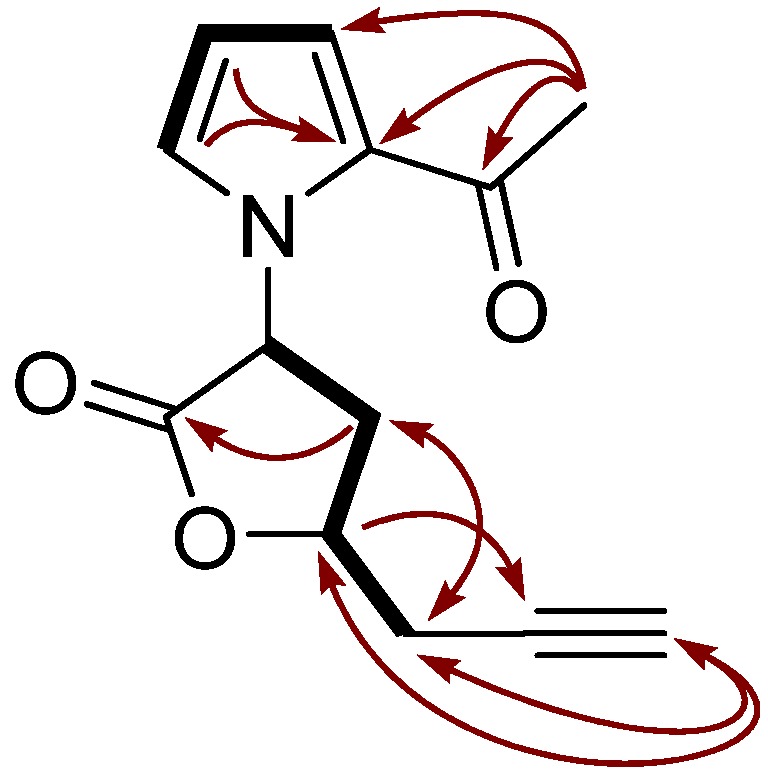
^1^H-^1^H COSY (bold line) and key HMBC correlation (arrow) in longanlactone.

The ^1^H-^1^H COSY and ^1^H-^13^C COSY spectra revealed the partial structures shown by bold lines in Figure 2. Further connectivities were deduced from the HMBC and NOESY spectra (Table 1). The HMBC correlations from H-7' to C-2', C-3', C-6' indicated the acetyl group was attached on C-2'. The HMBC correlations from H-3 to C-1 and C-2, indicated connectivity of C-2 to C-1 and C-3. The correlations from H-7 to C-5 and C-4, from H-4 to C-6, indicated connectivity of the propargyl to the lactone ring on C-4. Key HMBC correlations (arrows) of this compound are shown in Figure 2. 

**Figure 3:**
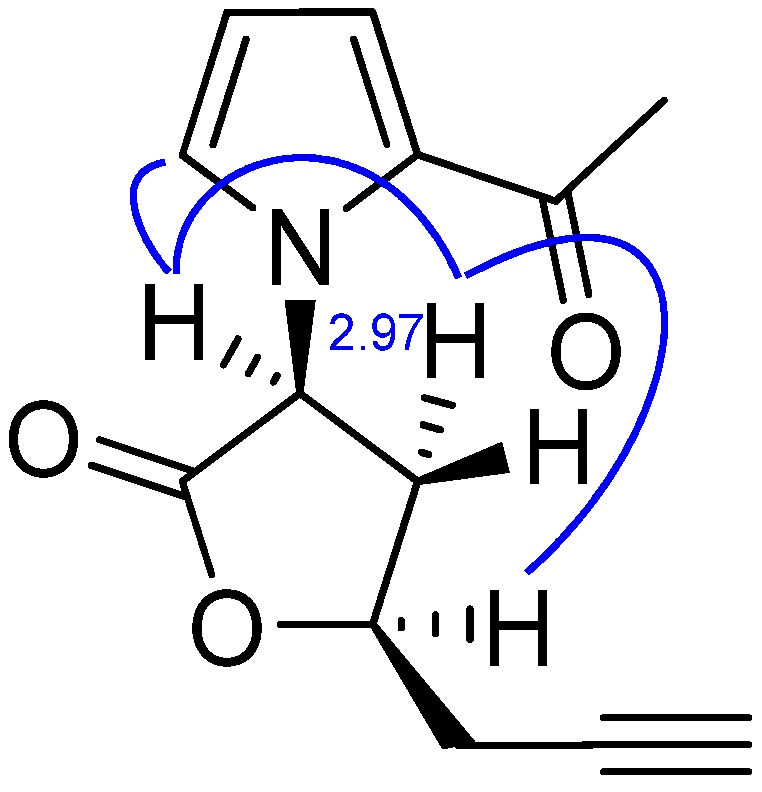
Key NOE correlations (curve) in longanlactone.

In the NOESY spectrum (Table 1 and Figure 3), the presence of mutual NOE correlations between H-5' and H-2 indicated the pyrrole ring and the lactone ring are connected through C-2 and the N atom. The mutual NOE correlations between H-2, H-3a and H-4 indicated that H-2 and H-4 are located on the same side of the lactone ring, so the relative configuration of this compound was established as depicted in Figure 3.

## 3. Experimental

### 3.1. General

Optical rotations were obtained on a Perkin-Elmer 343 spectropolarimeter (Perkin-Elmer, Boston, MA, USA). Melting points were determined with a micromelting point apparatus (Yanagimoto Seisakusho Ltd., Kyoto, Japan). UV spectra were recorded in MeOH on a Perkin-Elmer Lambda 25 UV-Vis spectrophotometer. ^1^H-NMR (400 MHz) and ^13^C-NMR (100 MHz) spectra were recorded on a Bruker DRX-400 instrument (Bruker BioSpin, Ettlingen, Germany) in CDCl_3_ with the residual solvent peak (δ_H_ 7.26 and δ_C_ 77.36) as reference. HRESIMS data were obtained on a Bruker Bio TOF IIIQ mass spectrometer (Bruker Daltonics, Billerica, MA, USA). ESIMS were collected on a MDS SCIEX API 2000 LC/MS/MS instrument (AB MDS Sciex, Toronto, Canada). For column chromatography, silica gel 60 (100−200 mesh, Qingdao Marine Chemical, Qingdao, China), and Sephadex LH-20 (Pharmacia Fine Chemicals, Uppsala, Sweden) were used. TLC was performed on precoated silica gel plates (GF_254_, Qingdao Marine Chemical) with detection under UV light (λ = 254 nm), exposure in I_2_ vapour, and spray of H_2_SO_4_ solution (10%) in EtOH, followed by heating.

### 3.2. Plant Material

Longan seeds were collected from a commercial longan orchard located in Maoming, Guangdong, China, in September 2005. The seeds were sun dried, and ground to powder.

### 3.3. Extraction and Isolation

The longan seed powder (10.5 kg), after defatting with petroleum ether, was extracted with 95% EtOH (150 L) three times at room temperature (24 h per time). The EtOH solutions were combined and concentrated under vacuum. The residue was suspended in H_2_O and partitioned successively with petroleum ether and CHCl_3_ (10 L, room temperature, 6 h) to obtain CHCl_3_-soluble extracts (39.0 g). The CHCl_3_-soluble extract was subjected to silica gel column chromatography (CC) and eluted with CHCl_3_-MeOH mixtures with increasing polarities (10:0–4:1) to obtain sixteen fractions A–P. Fraction D (31 mg), obtained on elution with 99:1 CHCl_3_–MeOH was further subjected to silica gel CC, eluted with petroleum ether-acetone mixtures with increasing polarities (99:1–9:1), to afford five subfractions D1–D5. Subfraction D4 was separated by CC on Sephadex LH-20 with MeOH as eluant to yield the title compound (3 mg).

### 3.4. Spectral Data

*Longanlactone*: white acicular crystals, m.p. 198–200 °C; positive HRESIMS *m/z* 232.0971 [M+H]^+^ (calculated for C_13_H_14_NO_3_, 232.0973); positive ESIMS *m/z* 232 [M+H]^+^, 254 [M+Na]^+^, 270 [M+K]^+^; [*α*]*_D_*^20^ -9° (C = 0.2 in acetone); UV: λ_max_ (*ε*) 202 (9867), 243 (sh. 3253), 253 (3541), 286 (5924); IR (KBr) ν_max_: 3306, 3130, 3116, 2992, 2926, 2111, 1770, 1640, 1548, 1486, 1037, 870, 775, 760 cm^−1^; ^1^H-NMR (400 MHz, CDCl_3_) and ^13^C-NMR (100 MHz, CDCl_3_) see Table 1.

## 4. Conclusions

In summary, the new compound longanlactone isolated from *Dimocarpuslongan* Lour. seeds was identified as 3-(2-acetyl-1*H*-pyrrol-1-yl)-5-(prop-2-yn-1-yl)dihydrofuran-2(3H)-one by its spectral data, including MS, and 1D and 2D NMR. 
